# Mapping disulfide bonds from sub-micrograms of purified proteins or micrograms of complex protein mixtures

**DOI:** 10.1007/s41048-018-0050-6

**Published:** 2018-04-23

**Authors:** Shan Lu, Yong Cao, Sheng-Bo Fan, Zhen-Lin Chen, Run-Qian Fang, Si-Min He, Meng-Qiu Dong

**Affiliations:** 1 National Institute of Biological Sciences, Beijing, Beijing, 102206 China; 2 Key Lab of Intelligent Information Processing of Chinese Academy of Sciences (CAS), University of CAS, Institute of Computing Technology, CAS, Beijing, 100190 China; 3 University of Chinese Academy of Sciences, Beijing, 100049 China

**Keywords:** Disulfide, Identification of disulfide bonds, Cross-linking, Mass spectrometry, PLink, PLink-SS

## Abstract

Disulfide bonds are vital for protein functions, but locating the linkage sites has been a challenge in protein chemistry, especially when the quantity of a sample is small or the complexity is high. In 2015, our laboratory developed a sensitive and efficient method for mapping protein disulfide bonds from simple or complex samples (Lu *et al*. in Nat Methods 12:329, 2015). This method is based on liquid chromatography–mass spectrometry (LC–MS) and a powerful data analysis software tool named pLink. To facilitate application of this method, we present step-by-step disulfide mapping protocols for three types of samples—purified proteins in solution, proteins in SDS-PAGE gels, and complex protein mixtures in solution. The minimum amount of protein required for this method can be as low as several hundred nanograms for purified proteins, or tens of micrograms for a mixture of hundreds of proteins. The entire workflow—from sample preparation to LC–MS and data analysis—is described in great detail. We believe that this protocol can be easily implemented in any laboratory with access to a fast-scanning, high-resolution, and accurate-mass LC–MS system.

## Introduction

### Functions of disulfide bonds

Formation of disulfide bonds is a common post-translational modification that has important biological functions. Many secreted proteins such as antibodies, growth factors, extracellular matrix proteins, and cell surface receptors or transporters, which happen to be of great therapeutic interest, are rich in disulfide bonds. As a structural building block, a disulfide bond covalently links two cysteine residues in the same protein or in different proteins to strengthen the correct conformation of a protein or protein complex, thereby improving stability. Further, the reversible nature of a disulfide bond enables it to act as a molecular switch to regulate the activity of enzymes or transcription factors in response to the redox state of the environment (Hogg 2003). To fully understand the biological function of a disulfide-containing protein and its regulation, it is necessary to map precisely the position of each disulfide bond and to determine the redox state of the two cysteine residues involved—in the disulfide form or as free thiols, or else—under conditions studied.

### Methods for disulfide bond analysis

In the past, a variety of methods had been used for the analysis of protein disulfide bonds including Edman degradation (Haniu *et al*. 1994), diagonal electrophoresis (McDonagh 2009), mutagenesis of cysteine residues coupled with reducing and non-reducing SDS-PAGE (Itakura *et al*. 1994), X-ray crystallography (McCarthy *et al*. 2000), and nuclear magnetic resonance spectroscopy (Sharma and Rajarathnam 2000). However, none of these methods are ideal; the ones that can provide precise linkage information of disulfide bonds demand highly specialized skills and devoted efforts of structural biologists, and the ones that can be executed in an average biology lab do not afford linkage information directly. Further, they usually require milligrams of purified proteins, and none of them work on complex samples.

In recent years, rapid technological development in liquid chromatography–mass spectrometry (LC–MS) has made it possible to map disulfide bonds from as little as micrograms of proteins in a relatively high throughput way (Choi *et al*. 2009; Götze *et al*. 2012; Huang *et al*. 2014; Liu *et al*. 2014, 2017a; Murad and Singh 2013; Wang *et al*. 2014; Wefing *et al*. 2006; Wu *et al*. 2008; Xu *et al*. 2007). Among the methods in this category, the more straightforward ones do not reduce disulfide bonds prior to LC–MS analysis, so the linkage information can be extracted from the fragmentation spectra of peptides containing disulfide bonds. Different fragmentation methods including collision-induced dissociation (CID), higher-energy collisional dissociation (HCD), electron-transfer dissociation (ETD), and electron-transfer/higher-energy collision dissociation (EThcD) (Liu *et al*. 2014) have been used to varying degrees of success. Table 1 summarizes the data analysis tools that have been developed for LC–MS analysis of disulfide bonds. Most of them are designed to identify disulfide bonds directly from fragmentation spectra. From these endeavors, two challenges have become apparent, one is to identify all the disulfide bonds in a protein and the other is to identify disulfide bonds at a proteome scale, that is, from highly complex samples such as cell lysates, isolated mitochondria, or secretomes. Another constant problem is false identification of disulfide bonds, the source of which may be faulty data analysis or disulfide bond scrambling during sample preparation.

**Table 1:** Software tools for MS-based disulfide bond identification

Software	MS function required	Type of data analyzed	Advantages (A) and limitations (L)	References
SearchXLinks	MALDI source	MS1	L: Works only for very simple samples	Wefing *et al*. (2006)
MassMatrix	CID or HCD	CID or HCD MS2	A: Complex forms of disulfide bonds are taken into consideration; L: Only for low-complexity samples digested using specific proteases	Xu *et al*. (2007)
DBond	CID or HCD	CID or HCD MS2	A: Disulfide-specific fragment ions are considered; L: No automatic FDR control, only for low-complexity samples	Choi *et* *al*. (2009)
MS2DB+	CID or HCD	CID or HCD MS2	L: No FDR control	Murad and Singh (2013)
MixDB	CID or HCD	CID or HCD MS2	A: Automatic FDR control, can handle large protein databases; L: Not easy to use, not tested with real-world samples	Wang *et al*. (2014)
RADAR	HCD	HCD MS2	A: Specific dimethyl labeling at peptide N-terminus improves accuracy of identification; L: Requires labeling after digestion; No FDR control	Huang *et al*. (2014)
PepFinder	EThcD or high-resolution ETD	HCD MS2	L: One reduced and one non-reduced samples need to be analyzed side by side, works only for low-complexity samples	From Thermo Scientific
SlinkS	EThcD and high-resolution ETD	ETD or EThcD MS2	A: ETD and EThcD complement each other; L: Requires a unique ion pattern, efficiency of ETD varies depending on the peptides	Liu *et al*. (2014)
pLink-SS	HCD or high-resolution ETD	HCD MS2	A: Automatic workflow with FDR estimation, disulfide-specific ions and internal ions are considered, works for complex samples; L: highly complex forms such as three peptides inter-linked through two disulfide bonds cannot be identified	Lu *et al*. (2015)

### About the method used in this protocol

**Fig. 1:**
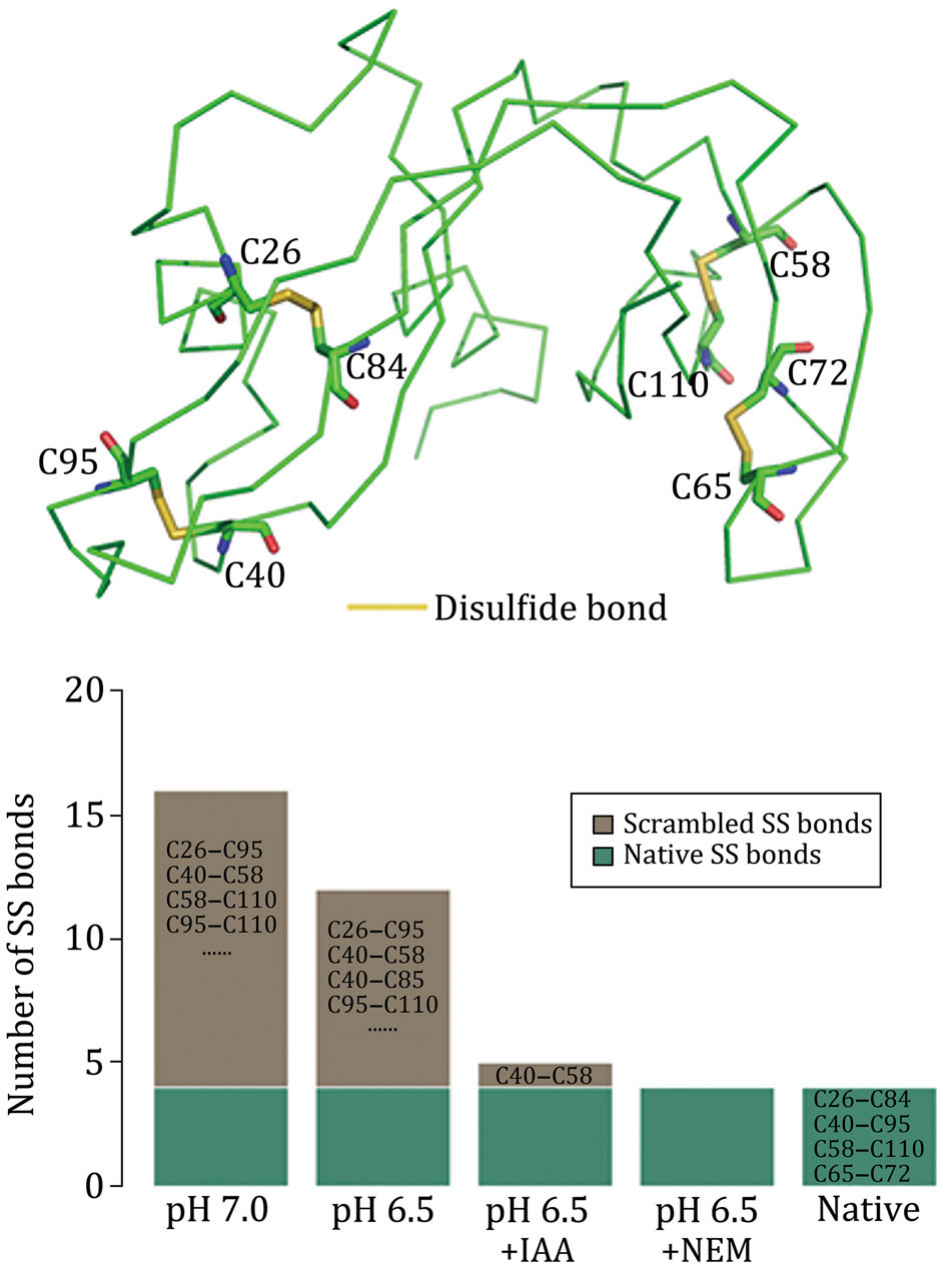
Non-native disulfide bonds of RNase A are abolished by blocking free thiols with NEM and carrying out protease digestions at pH 6.5

**Fig. 2:**
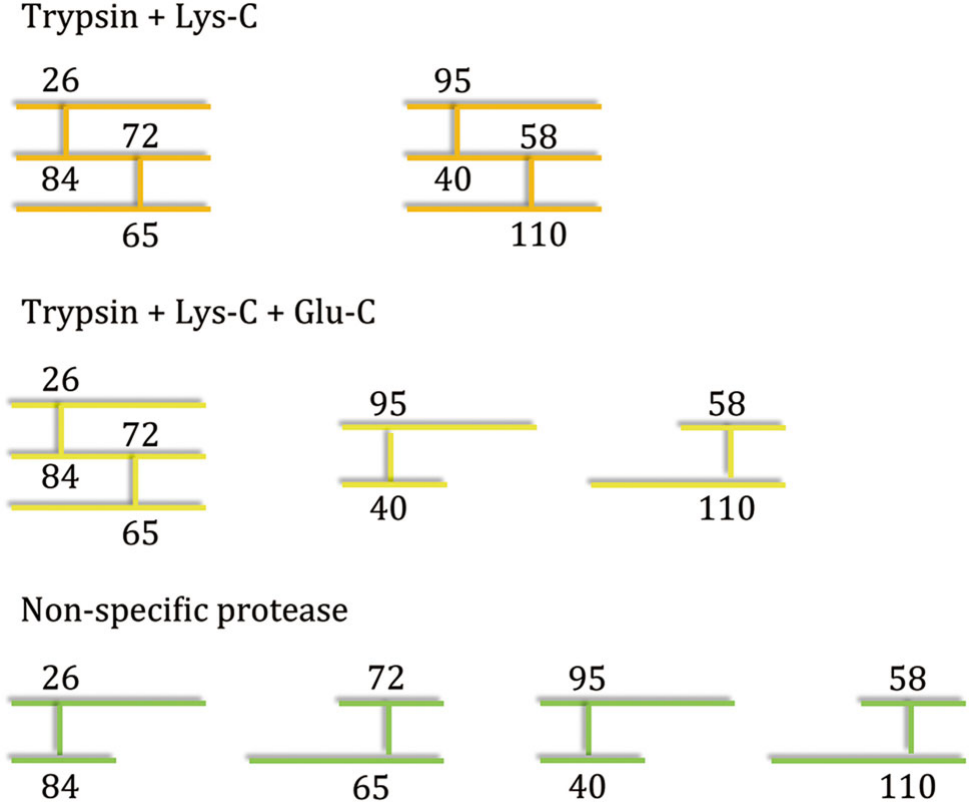
Digestion of RNase A with Lys-C and trypsin results in a complex form comprising three peptides linked together through two disulfide bonds, which cannot be identified using existing software tools. Digestion with additional proteases generates simpler forms that can be identified

**Fig. 3:**
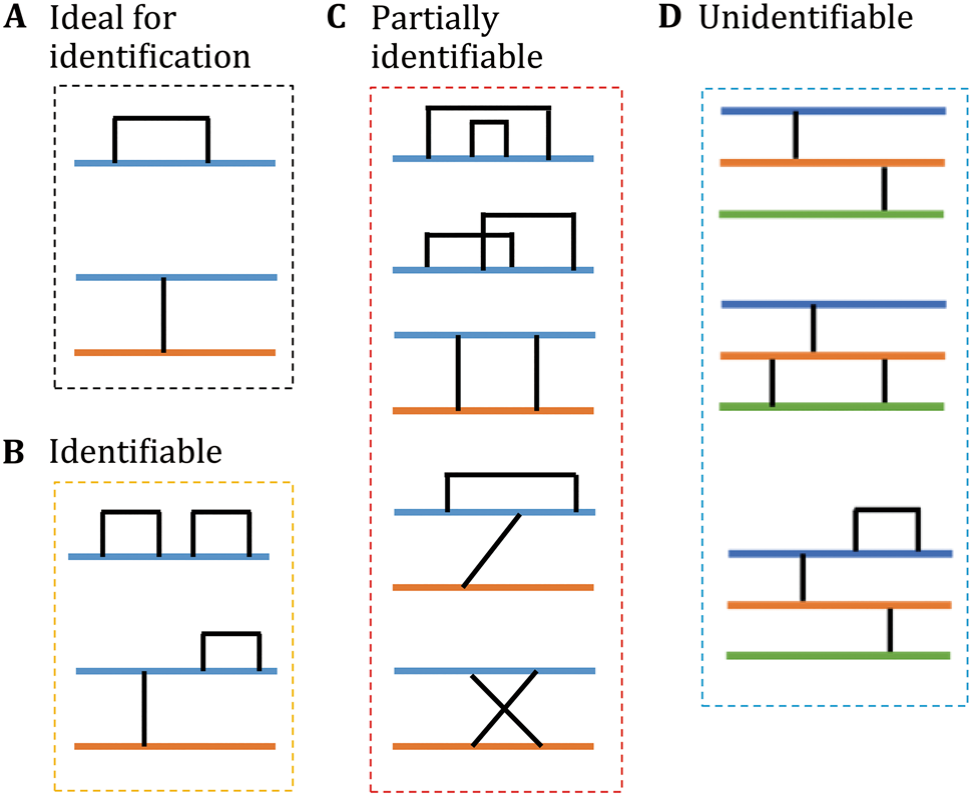
Disulfide bonds that can or cannot be identified using pLink. **A** Peptides containing a single disulfide bond can be identified with precise linkage information. **B** Disulfide bonds can be identified with precise linkage information in some cases. **C** The presence of disulfide bonds may be identified but without linkage information. **D** Disulfide bonds cannot be identified

With these problems in mind, we developed a method that enabled us to prevent most, if not all disulfide scrambling events and to identify all the native disulfide bonds of a single protein or a mixture of ten proteins from micrograms or even a few hundred nanograms of samples. It also enabled us to map native disulfide bonds at a proteome scale, for instance, 199 disulfide bonds were identified from a periplasmic fraction of *Escherichia coli* cells and 568 disulfide bonds were identified from proteins secreted by human umbilical vein endothelial cells. This method was published in 2015 (Lu *et al*. 2015). Since then, to our knowledge, it has been used successfully in dozens of studies and seven of them have been published (Hartman *et al*. 2016; Hung *et al*. 2016; Liu *et al*. 2017b; Mauney *et al*. 2017; Wang *et al*. 2016; Wu *et al*. 2016, 2017). The three key components of this method are as follows. First, disulfide bond scrambling is prevented by blocking free thiols with *N*-ethylmaleimide (NEM) and by maintaining an acidic pH throughout the sample preparation process, the latter of which includes precipitating freshly prepared protein samples with trichloroacetic acid (TCA) as early as possible and carrying out all protease digestions at pH 6.5 (Fig. 1). Second, to identify all the disulfide bonds of a protein, multiple proteases are utilized, even the non-specific ones such as proteinase K. This is because some disulfide bonds may be present in a complex form that is difficult to identify if the sample is digested only with Lys-C and trypsin (Fig. 2). Last and the most important, a data analysis program called pLink-SS has been developed and carefully tuned to identify disulfide-bonded peptides from HCD spectra. The types of disulfide-bonded peptides that can be identified using pLink-SS are shown in Fig. 3. Presently, pLink-SS has been incorporated into pLink 2, which is an upgraded version of pLink and remains free for academic users. pLink 2 is ~40 times faster than pLink, with a friendly graphical interface and some further improvements in accuracy. pLink 2 was officially released on January 1, 2018 and can be downloaded at http://pfind.ict.ac.cn/software/pLink/.

**Fig. 4:**
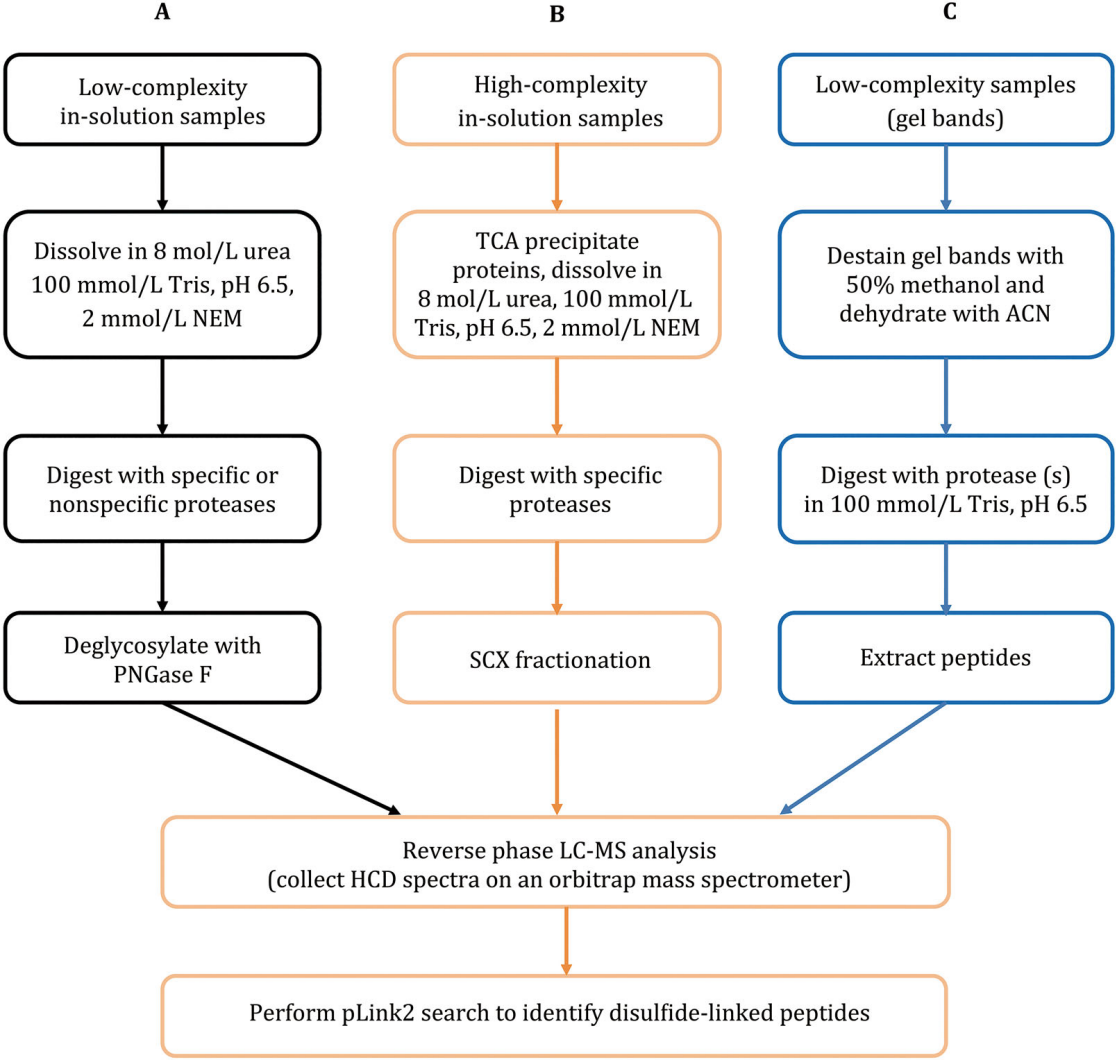
Flowchart of this protocol

In this paper, we present a step-by-step disulfide mapping protocol using the method we developed in 2015. As shown in Fig. 4, this protocol contains three alternative sub-protocols that are each optimized for low-complexity samples in solution, protein gel bands, or high-complexity samples.

## Reagents

### Chemicals

- Acetic acid (J. T. Baker, cat. no. 9508)
- Acetone (J. T. Baker, cat. no. 9002-02)
- Acetonitrile (ACN), HPLC grade (Fisher Scientific, cat. no. A998)
- Ammonium acetate, 7.5 mol/L solution (Sigma-Aldrich, cat. no. A2706)
- BCA protein assay kit (Pierce, cat. no. 23228)
- Formic acid (FA) (J. T. Baker, cat. no. 0129)
- Frit kit (including formamide, Kasil^®^-1 and Kasil^®^-1624 potassium silicate solution and a ceramic tubing cutter, from Next Advance)
- Guanidine hydrochloride (GndCl) (Sigma-Aldrich, cat. no. G3272)
- N-ethylmaleimide (NEM) (Pierce, cat. no. 23030)
- Trichloroacetic acid (TCA), 6.1N or 100% (*w*/*v*) solution (Sigma-Aldrich, cat. no. T0699)
- Tris (Amresco, cat. no. 0497)
- Urea (Sigma-Aldrich, cat. no. U0631)
- Water, HPLC grade (Sigma-Aldrich, cat. no. V270733)

### Enzymes

- Asp-N (Promega, cat. no. V162A)
- Elastase (Sigma-Aldrich, cat. no. E7885)
- Glu-C (Promega, cat. no. V1651)
- Lys-C (Wako, cat. no. 125-05061)
- PNGase F (NEB, cat. no. P0704)
- Proteinase K (Sigma-Aldrich, cat. no. P3910)
- Subtilisin (Sigma-Aldrich, cat. no. 43538)
- Trypsin (Promega, cat. no. V5117)

### Supplies

- Fused silica capillary tubing with a polyimide coating for reverse-phase (RP) liquid chromatography–mass spectrometry (LC–MS), 75-μm inner diameter (ID), 360-μm outer diameter (OD) (Polymicro Technologies, Part No. 1068150019)
- Fused silica capillary tubing for off-line SCX fractionation, 200-μm ID, 360-μm OD (Polymicro Technologies, Part No. 1068150204)
- MicroTight Union for 360-μm OD tubing (Upchurch, Part No. P-772)
- Luna C18 resin, 3 μm particle size, 100 Å pore size (Phenomenex, cat. no. 04A-4251)
- Luna SCX resin, 5 μm particle size, 100 Å pore size, (Phenomenex, cat. no. 04A-4398)
- Sample vials for LC–MS (Thermo La-pha-pak, cat. no. 11190933)
- Welch Ultimate UHPLC-XB-C18 resin, C18, 1.8 μm particle size, 120 Å pore size (Welch Materials, Shanghai, cat. no. UHP101.02)
- YMC*GEL, C18, 10 μm particle size, pore size 120 Å (YMC, cat no. AQ12S11)

## Buffers

- 0.5 mol/L Tris buffer, pH 6.5
- Strong cation exchange (SCX) elution buffers: 25, 50, 100, 250, 500 mmol/L, and 1 mol/L ammonium acetate in 5% ACN, 0.1% FA
- For SCX fractionation: 80% ACN, 0.1% FA
- For in-gel digestion, peptide extraction (Buffer I): 0.5% FA, 50% ACN
- For in-gel digestion, peptide extraction (Buffer II): 1% FA, 75% ACN
- LC mobile phase A (Buffer A): H_2_O/FA (100/0.1, *v*/*v*), good at room temperature (RT) for several weeks
- LC mobile phase B (Buffer B): ACN/FA (100/0.1, *v*/*v*), good at RT for several months

## Equipment

### Small equipment

- ThermoMixer, a bench top temperature-controlled tube mixer (Eppendorf)
- Refrigerated bench top centrifuge (Eppendorf)
- SpeedVac™ concentrator (Fisher Scientific)
- A common laboratory oven or dryer
- Laser-based micropipette puller, model P-2000 (Sutter Instrument)
- Column packing setup, consisting of a pressure injection cell (also known as pressure loading cell or bomb loader) model PC77-MAG (Next Advance), a high-purity nitrogen gas canister (from a local supplier) fitted with a high-pressure regulator, and a stainless steel 1/8-inch diameter tubing that connects the regulator to the pressure injection cell. The last two items are parts of a column packing kit (Next Advance)

### LC–MS system

- Easy-nLC1000 liquid chromatography system (Thermo Fisher Scientific) or a similar nano-flow (200–600 nl/min) HPLC system with an autosampler
- Q-Exactive™ Q-Orbitrap mass spectrometer (Thermo Fisher Scientific) or a similar fast-scanning, high-resolution, accurate-mass MS instrument that can collect ten or more high-resolution (*R* > 7000) MS2 spectra per second

### Do-it-yourself capillary chromatography columns

Needless to say, skip this section if one chooses to purchase pre-packed columns of equivalent properties.

#### RP analytical column with a spray tip

i. Cut a 50-cm-long 75-μm ID fused silica tubing, burn the center segment (2–3 cm) over an ethanol burner, and wipe the blackened coating off the tubing with a sheet of kimwipe moistened with methanol.
ii. Mount the tubing into the Sutter laser puller with the clear segment in the path of the laser beam and pull a 5-μm tip.
  **Note**: The exact setup of the pulling program varies between instruments and over time. The example below serves as a starting point for optimization.
  cycle 1: HEAT = 270, FIL = 0, VEL = 35, DEL = 128, PUL = 0
  cycle 2: HEAT = 260, FIL = 0, VEL = 30, DEL = 128, PUL = 0
  cycle 3: HEAT = 250, FIL = 0, VEL = 25, DEL = 128, PUL = 0
  cycle 4: HEAT = 240, FIL = 0, VEL = 25, DEL = 128, PUL = 0
iii. Dismount the two empty columns each with a pulled tip.
iv. In a tube or a glass vial that will fit inside the pressure injection cell, add a small amount (roughly the size of a grain of millet) of Welch UHPLC-XB-C18 resin into methanol and make a slurry.
v. Using the column packing setup, pack the 3-μm Luna C18 resin into a 75-μm ID analytical column with a pulled tip for a length of ~2 cm, then switch the resin to 1.8-μm Welch UHPLC-XB-C18, and pack another 10–12 cm. The total length of the reverse phase is 13 ± 1 cm.
vi. Condition the column by running a RP gradient through it and ending with Buffer A wash.

#### RP trap column

i. Take from the Frit kit 60 μl Kasil-1624 and 20 μl Kasil-1, mix well in a small tube, then add 20 μl formamide, mix well.
ii. Cut a 20-cm-long 75-μm ID fused silica tubing, dip one end into the mixture just made, and then pull out immediately. Inspect the column for the appearance of a segment of liquid inside.
iii. Place the column in an oven of 100 °C for 4–12 h to obtain a porous frit at one end of the column. Before polymerization, handle the column with great care to avoid displacing the liquid away from the end. Keep fritted columns at RT for long-term storage.
iv. Before packing a column, cut the frit end with a tubing cutter so only 1–2 mm frit is left.
v. In a tube or a glass vial that will fit inside the pressure injection cell, add a small amount (roughly the size of a grain of millet) of Luna C18 resin into methanol and make a slurry.
vi. Using the column packing setup, pack the 10-μm YMC*GEL C18 resin into an empty, 75-μm ID column against the frit for a length of 7 ± 1 cm.
vii. Condition the trap column by passing Buffer A through it.

#### SCX fractionation column

i. Take from the Frit kit 60 μl Kasil-1624 and 20 μl Kasil-1, mix well in a small tube, then add 20 μl formamide, mix well.
ii. Cut a 25-cm-long 200-μm ID fused silica tubing, dip one end into the mixture just made, and then pull out immediately. Inspect the column for the appearance of a segment of liquid inside.
iii. Place the column in an oven of 100 °C for 4–12 h to obtain a porous frit at one end of the column. Before polymerization, handle the column with great care to avoid displacing the liquid away from the end. Keep fritted columns at RT for long-term storage.
iv. Before packing a column, cut the frit end with a tubing cutter so only 1–2 mm frit is left.
v. In a tube or a glass vial that will fit inside the pressure injection cell, add a small amount (roughly the size of a grain of millet) of Luna SCX resins into methanol and make a slurry.
vi. Using the column packing setup, pack the SCX resins into an empty column against the frit for a length of 2–3 cm.
vii. Pass Buffer A through the column to pack the SCX segment more tightly.
viii. Change the packing material to 3-μm Luna C18 resin and pack a RP segment of 2–3 cm.
ix. Condition the SCX column by passing Buffer A through it.

## Software

- Computer workstation with Microsoft Windows 7 or a newer operating system
- NET framework 4.5
- MSFileReader, both 32 and 64-bit version, for pLink 2 to access the raw file
- pLink 2, version 2.3.0
- Python 3.6

## Reagent setup

### 1 mol/L NEM stock solution

Dissolve 125.1 mg of solid N-ethylmaleimide in 1 ml of 100% ACN, dispense into 4-µl aliquots, store in a desiccator at −20 °C, and use within two months. Each aliquot is for a single use.

### 8 mol/L urea in 100 mmol/L Tris pH 6.5

Dissolve 240 mg of solid urea in 100 µl 0.5 mol/L Tris at pH 6.5 and 220 µl H_2_O. Prepare the fresh solution each time to minimize urea degradation and subsequent carbamylation of proteins or peptides.

### Stock solutions of protease*s*

Prepare 0.5 μg/μL trypsin or a different protease in H_2_O or a stock buffer specified by the vendor, dispense into aliquots of 10 µl or less, and store at −80 °C. Ideally, each aliquot is for a single use.

## Equipment setup

### LC–MS

On Q-Exactive, generate a MS method as follows: spray voltage 2.0–2.3 kV, data-dependent mode, full scan resolution 140,000, MS2 scan resolution 17,500, isolation window 2.0 *m/z*, AGC target at 1e6 for FTMS full scan and 5e4 for MS2, minimal signal threshold for MS2 at 4e4; normalized collision energy at 27%; peptide match preferred, and HCD spectra were collected for the ten most intense precursors carrying +3, +4,…, or +7 positive charges, dynamic exclusion 60 s. To increase the identification of loop-linked disulfide bonds, a technical repeat run is recommended in which +2 precursors were also included. So, generate another MS method that is the same as above except that +2 precursors are not excluded.

On Easy-nLC 1000 UHPLC, set the sample loading and RP gradient method as follows. For each sample, 0.5 µg of digested peptides are loaded onto the trap column at 1 µl/min and desalted with 10 µl of Buffer A. The peptides are separated through a 100 min linear gradient from 100% Buffer A to 30% Buffer B and then going up to 100% Buffer B in 1 min, followed by a 3-min 100% Buffer B wash before returning to 100% Buffer A in 2 min and maintaining at 100% Buffer A for 4 min. Set the flow rate at 250 nl/min.

## Software setup

1. MSFileReader: Instructions are available at https://github.com/pFindStudio/pLink2/wiki/FAQ#how-to-install-msfilereader.
2. pLink 2: Free download at http://pfind.ict.ac.cn/software/pLink/index.html#Downloads. Double click the installation package, choose the language and directory. pLink 2 will then finish the installation automatically.
  **[CRITICAL]** License is required to run pLink 2 the first time. To receive the license file, send an e-mail to pLink@ict.ac.cn. Follow instructions during installation. The pLink 2 installation package includes pParse, a MS data conversion tool, and pLabel—a very convenient and powerful tool for annotating peaks in a MS2 spectrum. Once pLink 2 is installed, pParse and pLabel are ready.
3. Python 3.6: Installation instructions are available at https://www.python.org/.
4. Python package “openpyxl” and “xlrd”: Enter in the following commands in windows “Command Prompt”:
  pip3 install openpyxl,
  pip3 install xlrd.
  **[CRITICAL]** Here we provide a python script to organize the pLink 2 search results and generate a simple and informative report file. To run this script, Python 3.6 and packages openpyxl and xlrd are required.
5. Python script SS_sim.py: Download from https://github.com/daheitu/pLink2_ss_results_analysis.github.io.git.

## Sample preparation

### In-solution digestion (low-complexity samples) [TIMING ~ 1 day]

**Note:** Low-complexity samples refer to a purified protein or a protein complex of no more than 50 subunits.

1. Determine the concentration of a freshly purified protein sample using a BCA protein assay kit. Do not rely on *OD*_280_ measurements.
2. Based on the sequences of the proteins in question and the amount of proteins available, decide which proteases to use and how many digestions to carry out. Common choices are Lys-C/trypsin, Lys-C/trypsin/Glu-C, Lys-C/Asp-N, and Lys-C/trypsin/Asp-N; and additional options include Lys-C/elastase, subtilisin, and proteinase K.
3. For each digestion, take 4 μg of a freshly prepared protein sample and precipitate with 25% TCA. Specifically, add 1/3 volume of 100% TCA, mix well, and leave on ice for 30 min to overnight. Spin at 4 °C in a bench top centrifuge at top speed for 30 min to pellet proteins, wash with 0.5 ml cold acetone twice, and air dry the pellet.
4. Dissolve 4 μg of the freshly precipitated protein sample in 10 μl of 8 mol/L urea, 100 mmol/L Tris, pH 6.5; add NEM to a final concentration 2 mmol/L; and incubate at 37 °C for 2 h.
  **[CRITICAL]** NEM can gradually hydrolyze in water and lose activity, so 15 min before use, transfer a frozen aliquot of 1 mol/L NEM to a desiccator at RT.
**[? TROUBLESHOOTING]**
5. Digest the protein(s) with one or more proteases of choice. The digestion conditions of various proteases are listed in Table 2. If two or more proteases are to be combined in one digestion, perform the digestion sequentially to reduce mutual digestion between proteases. For example, if a sample is to be digested with Lys-C/trypsin/Glu-C, digest with Lys-C first in 8 mol/L urea at 37 °C for 2 h; then dilute to 2 mol/L urea with 30 μl of 100 mmol/L Tris, pH 6.5, add trypsin, and incubate at 37 °C for 12 h; and lastly, dilute to 1 mol/L urea with 40 μl of 100 mmol/L Tris, pH 6.5, add Glu-C, and incubate at 37 °C for 12 h.

**Table 2:** Proteases digestion conditions

	Lys-C	Trypsin	Glu-C	Asp-N	Elastase	Subtilisin	Proteinase K
Denaturant	8 mol/L urea	2 mol/L urea	1 mol/L urea	2 mol/L urea	2 mol/L urea	2 mol/L GndCl	2 mol/L GndCl
Digestion time	4 h	12 h	12 h	12 h	8 h	4 h	4 h
Enzyme: Protein (*w*:*w*)	1:100	1:20	1:40	1:50	1:20	1:20	1:20

Digestion temperature is 37 ºC for all
  **[CRITICAL]** As disulfide-linked proteins are often resistant to proteases, digestion in the presence of a high concentration of denaturant is necessary. Therefore, Lys-C digestion in 8 mol/L urea usually precedes other proteases except for subtilisin and proteinase K, both of which have activity high enough for digesting any protein, even at pH 6.5. Avoid under- or over-digestion.
**[? TROUBLESHOOTING]**
6. To remove glycosylation, which may interfere with data analysis, add PNGase F (112 NEB units per 6 µg of proteins) to the digest and incubate at 37 °C for 2 h.
  **[CRITICAL]** Many secreted proteins are glycosylated and disulfide-linked. Unexpected glycosylation prevents identification of disulfide bonds. PNGase F remains its activity in 2 mol/L urea or 1 mol/L GndCl but loses most of its activity in 2 mol/L GndCl, so dilute the proteinase K digest with an equal volume of 100 mmol/L Tris, pH 6.5 before adding PNGase F.
7. Quench the reaction by adding 90% FA to a 5% final concentration.
  For LC–MS analysis of a low-complexity sample, load 0.2–0.5 µg of protein for a single run.
  **[PAUSE POINT]** The samples can be stored up to several weeks at −20 °C or −80 °C before LC–MS analysis.

### In-solution digestion (high-complexity samples) [TIMING 1–2 days]

**Note:** High-complexity samples refer to whole-cell lysates, subcellular fractions, or crude immunoprecipitated proteins that contain hundreds or thousands of proteins.

1. Precipitate 30 μg of a freshly prepared protein sample with 25% TCA and wash with cold acetone as described above. For details, see “**In-solution digestion (low-complexity samples)**”.
2. Dissolve precipitated proteins in 25 μl of 8 mol/L urea, 100 mmol/L Tris, pH 6.5; add NEM to a final concentration of 2 mmol/L; and incubate at 37 °C for 2 h.
  **[CRITICAL]** Sonication is recommended to help dissolve proteins in 8 mol/L urea. 20–40 µg proteins are acceptable.
3. Digestion with Lys-C/trypsin/Glu-C. For details, see “**In-solution digestion (low-complexity samples)**”.
  **[CRITICAL]** Avoid using proteases of poor specificity to digest high-complexity samples as it will lead to search space explosion in data analysis, reducing the speed and sensitivity of identification. In our experience, adding Glu-C on top of Lys-C/trypsin digestion significantly increases the number of disulfide bond identifications. If desired, Lys-C/trypsin/Asp-N is another option.
4. Quench the digestion by adding FA to a final concentration of 5%.
5. Using a pressure injection cell, load digested peptides onto a SCX fractionation column. For better flow-rate control, connect the fritted end of the SCX column to an empty 75-μm ID, 360-μm OD fused silica tubing (with a pulled tip if necessary) with a MicroTight union. Wash the column with 15 μl of 0.1% FA, followed by 15 μl 80% ACN, 0.1% FA, then 10 μl of Buffer A, at a flow rate of 1 μl/min. Now the peptides are bound to SCX resin and ready to be fractionated.
6. Elute sequentially with 20 μl of 5% Buffer B (5% ACN, 0.1% FA) containing 25, 50, 75, 100, 500, or 1000 mmol/L ammonium acetate, pH 2–3, at a flow rate of 1.0–2.0 μl/min. Collect each of the six fractions into an Eppendorf tube. Load one-fifth of each fraction for a subsequent reverse-phase LC–MS run.
  **[PAUSE POINT]** The samples can be stored up to several weeks at −20 °C or −80 °C before LC–MS analysis.

### In-gel digestion (protein bands of interest) **[TIMING 1–2 days]**

**[CRITICAL]** To maintain protein disulfide bonds during SDS-PAGE analysis, reducing reagents are forbidden and 20 mmol/L NEM must be present in the sample loading buffer.

1. Excise the gel band of interest and dice into 1 mm^3^ pieces.
2. Destain the gel with 50% methanol and wash with ddH_2_O twice. Then dehydrate with 100% acetonitrile.
3. Rehydrate into 100 mmol/L Tris, pH 6.5 containing 0.5 mmol/L NEM, 5 ng/µL Lys-C, and 10 ng/µL of another protease of choice—trypsin, Glu-C, or Asp-N.
4. Digest for 12 h at 37 °C.
5. Extract peptides with 50–100 µl of extraction Buffer I (50% ACN, 0.5% FA) and then with 50–100 µl of extraction Buffer II (75% ACN, 1% FA).
6. Concentrate the sample to 4–8 µl in a SpeedVac™ concentrator at 2.5 Torr, RT. If the sample dries out accidently, reconstitute the sample with 6 µl of 0.1% FA, 1% ACN. Calculate or estimate the amount of protein in the gel band and prepare to load about 0.2 µg for LC–MS analysis. If this is not practical, load 1/5 of the sample.
  **[PAUSE POINT]** The samples can be stored up to several weeks at −20 °C or −80 °C before LC–MS analysis.

## LC–MS analysis [**TIMING ~ 1–30 h, depending on sample complexity]**

**[CRITICAL]** Each sample is to be analyzed twice; reject 2+ precursor ions in one (to increase identification of inter-linked peptides) but not in the other (to increase identification of loop-linked peptides).

1. Connect the trap column and the analytical column to an Easy-nLC 1000 UHPLC according to the 2-column setup scheme. Cut the tail end of each column to reduce dead volumes.
2. Connect the 2-column setup to the nano-ESI source of the Q-Exactive™ mass spectrometer and position the tip of the analytical column ~0.5 cm away from the opening of the heated capillary. Adjust the tip to ensure a steady spray.
3. Pre-equilibrate the columns with Buffer A and make sure there is no air bubble.
4. Transfer samples to be analyzed into sample vials.
5. Place the sample vials into the autosampler of an Easy-nLC 1000 UHPLC.
6. Set up a method for each sample. Specify the names of the data files to be generated and the directory in which they will be stored. Load the LC and the MS methods written above (see “**Equipment setup**” for details).
7. Start the LC–MS analysis and monitor it from time to time till it finishes.

## Data analysis **[TIMING ~ 0.2–30 h]**

### Start a task

(8) Double click the pLink 2 icon on desktop or the pLink installation folder to start pLink 2. Click “New…” to start a new task, change the task “Name” and choose “Location”, then click “OK”.

### Import .raw files and set up the extraction parameters (Fig. 5)

(9) In the “MS data” panel, choose the “MS Data Format”. “RAW” is the default data format.

**Fig. 5:**
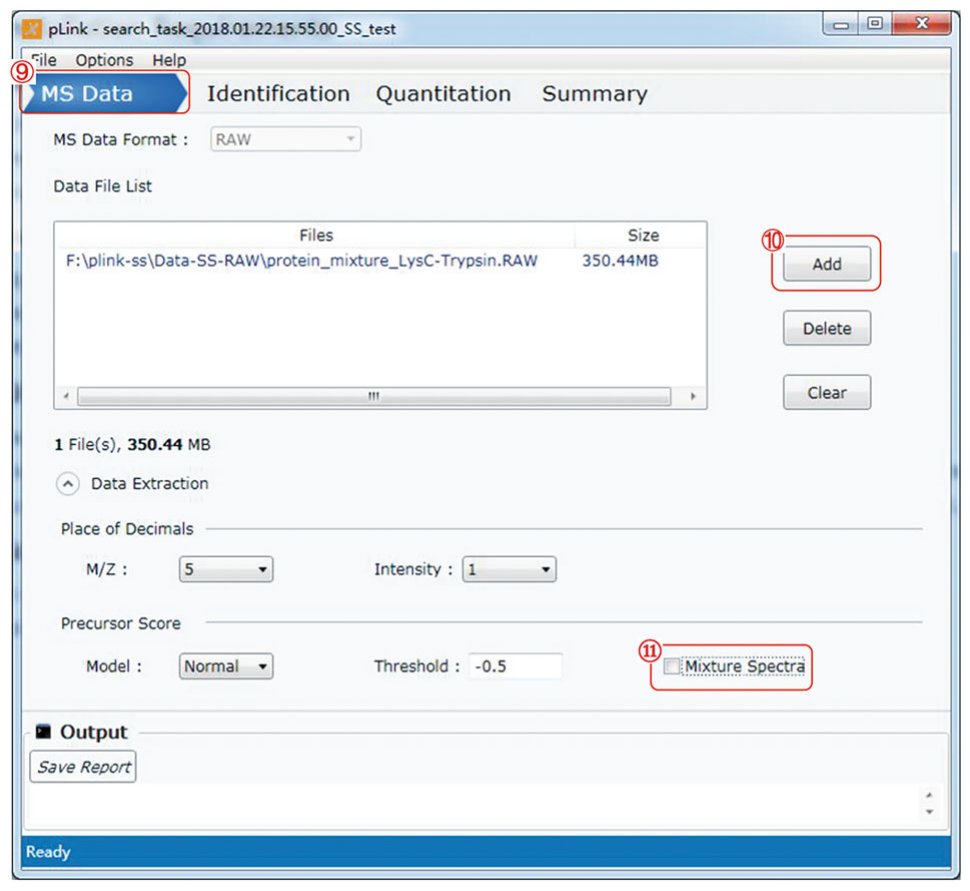
Import raw file and set extraction parameters
  **[CRITICAL]** Although pLink 2 can import “.mgf” files, we recommend the use of “.raw” files as input. The “.mgf” files extracted using other software tools may not be supported by pLink 2, and “.ms2” files are not allowed.
(10) Click the “Add” button, choose the input file(s).
(11) Make sure to uncheck “Mixture Spectra”. The default setting is on, but for disulfide bond identification it is better to turn it off.

### Set up the identification parameters (Fig. 6)

**Fig. 6:**
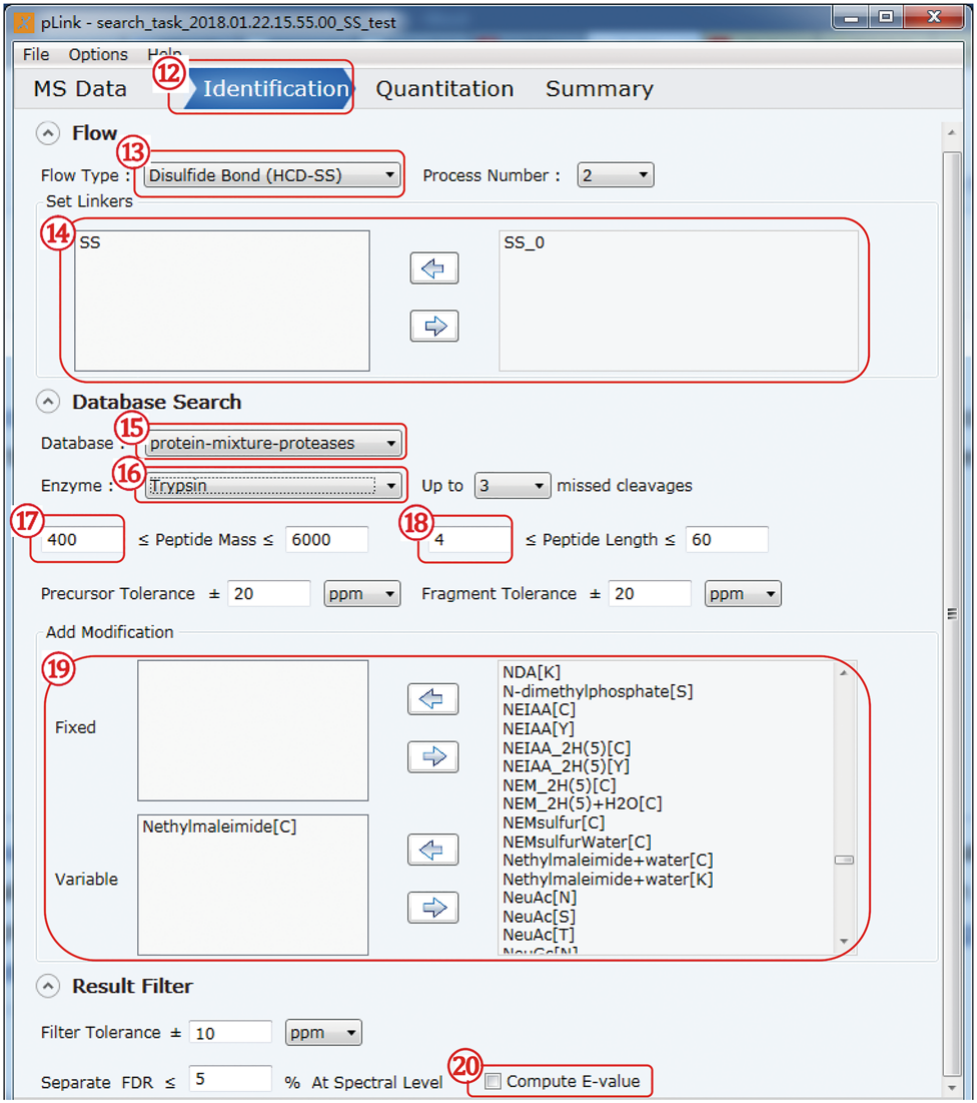
Set up the identification parameters

(12) Click to switch to the “Identification” panel.
(13) For “Flow Type”: choose “Disulfide bond (HCD-SS)”.
(14) In the “Set linkers” box, choose “SS” on the right side and click the arrow pointing to the left to transfer “SS” to the left side.
(15) Choose the database to use; you can add a new “fasta” file through the “Customize Database” option.
  **[CRITICAL]** we recommend that you append the database of common contaminant proteins stored in pLink 2 to your “fasta” database file.
(16) Select the enzymes that had been used to digest the samples, for example, “Trypsin” for Lys-C/trypsin digestion, “Glu-C.Trypsin” for Lys-C/trypsin/Glu-C digestion, and “non-specific” for Lys-C/elastase or protease K digestion.
(17) Change the “mini peptide mass” to 400.
(18) Change the “mini peptide length” to 4.
(19) Select “N-Ethylmaleimide[C]” on the right side and transfer it to the “Variable” modification box on the left.
(20) Check “Compute *E* value” to output *E* value for each PSM.
  **[CRITICAL]** pLink 2 uses a machine learning algorithm (SVM) to classify the target and decoy MS/MS spectra, and it only provides SVM-scores by default. Computing an *E* value for each PSM is highly desirable.

### Check all the parameter settings and start search (Fig. 7)

(21) Once the search parameters are complete, switch to the “Summary” panel.
(22) Double check the parameters. Go back and reset if anything is wrong.
(23) After verifying the parameters, click “Save” which will activate the “Start” button.
(24) Click “Start” and now the search begins.
(25) View results. When the “Output” panel displays “[pLink] Complete report”, the html results will be shown in your default browser (IE or chrome) automatically. You can also view the “csv” format results in the “reports” folder of pLink 2.
(26) To generate a concise summary of the disulfide-linked sites identified by pLink 2, copy the python script (attached to the end of this protocol) to your task folder, then open “Windows Explorer”, type “cmd” in the location bar, and press “Enter” to open “Command Prompt”. Lastly, enter the following in the command line:
  python SS_sim.py.

**Fig. 7:**
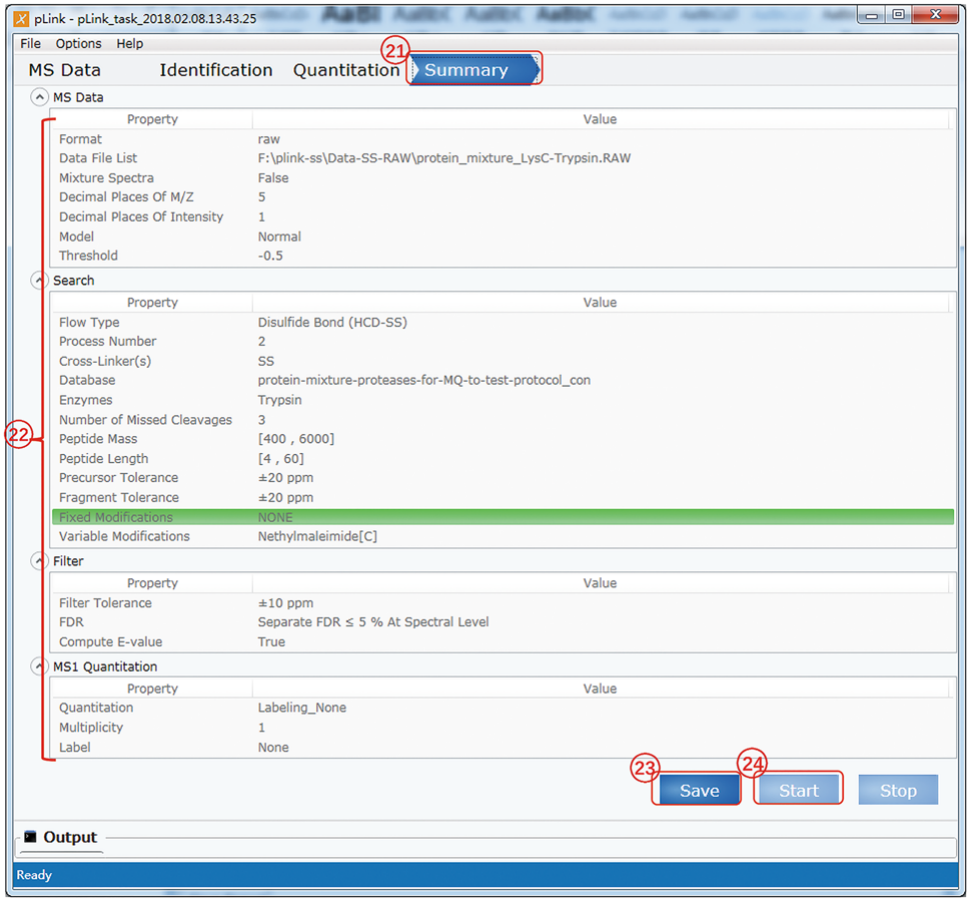
Summary and starting to search

## View ms/ms spectra using pLabel

### Load pLabel file (Fig. 8)

**Fig. 8:**
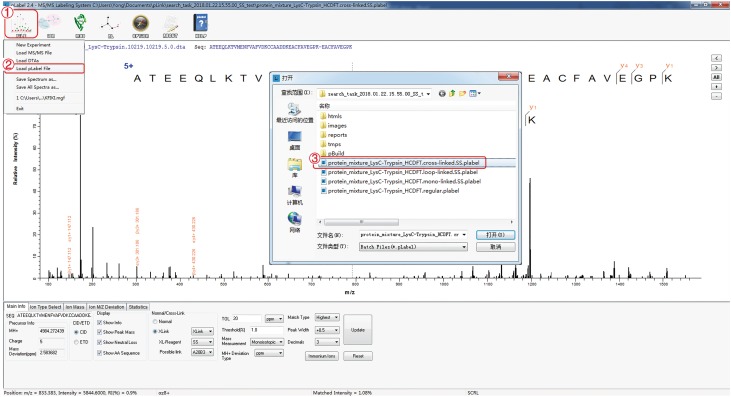
Load pLabel file

1. Double click the “pLabel” icon on desktop to open the window shown in Fig. 8. Click “FILE”.
2. Choose “Load pLabel File”.
3. Choose the “cross-linked.SS.plabel” file generated after a pLink 2 search.
**[? TROUBLESHOOTING]**

### Find the spectra of interest (Fig. 9)

(5) Click “All”.
(6) In the pop-up window, type in the scan number of interest, for example “3290”, in the “Key Word” field.
(7) Click “Search”.
(8) Choose the spectrum shown at the top.
(9) Click “OK”.

**Fig. 9:**
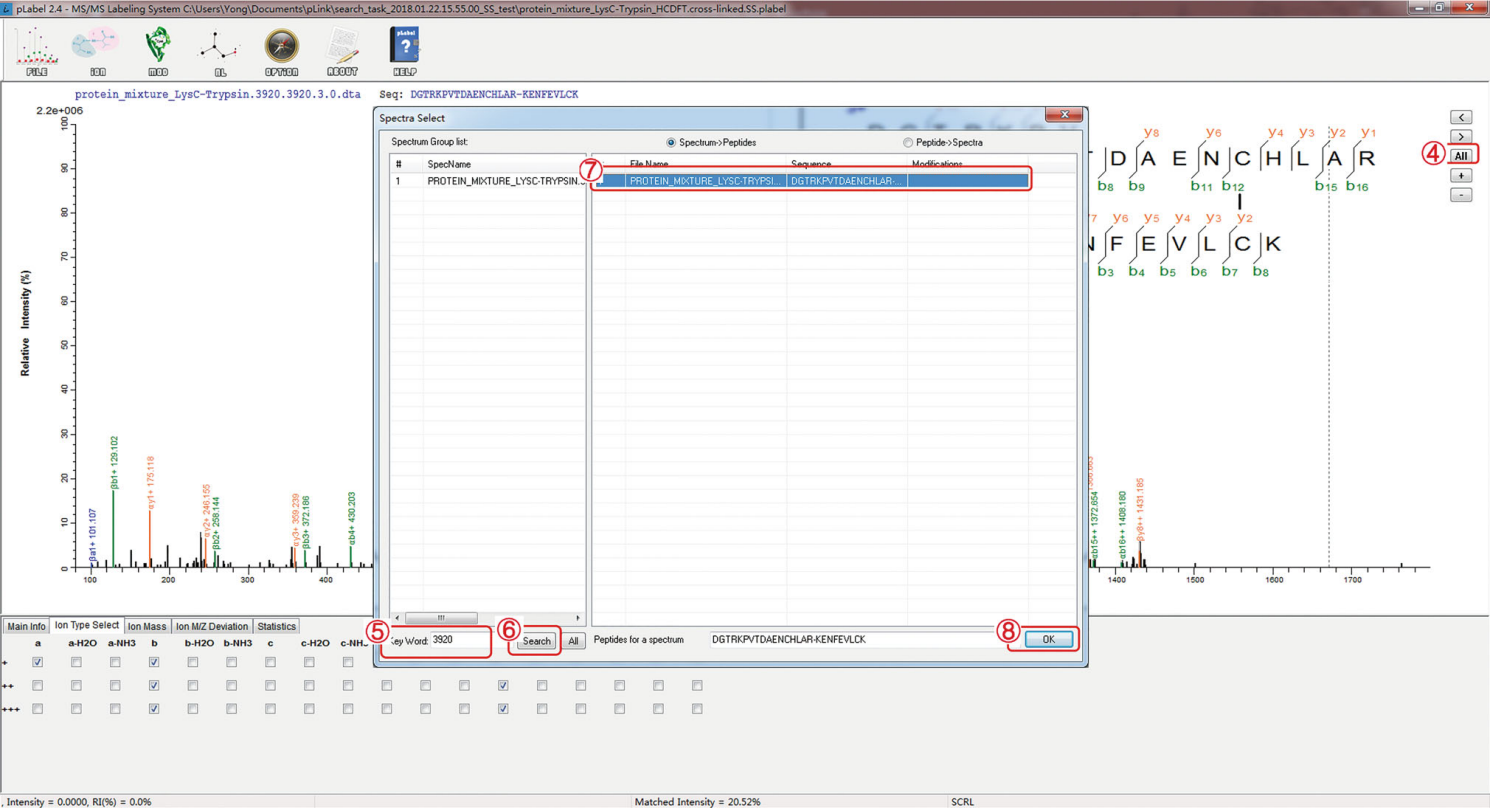
Find a representative spectrum of a disulfide-linked peptide or peptide pair for manual inspection

**Fig. 10:**
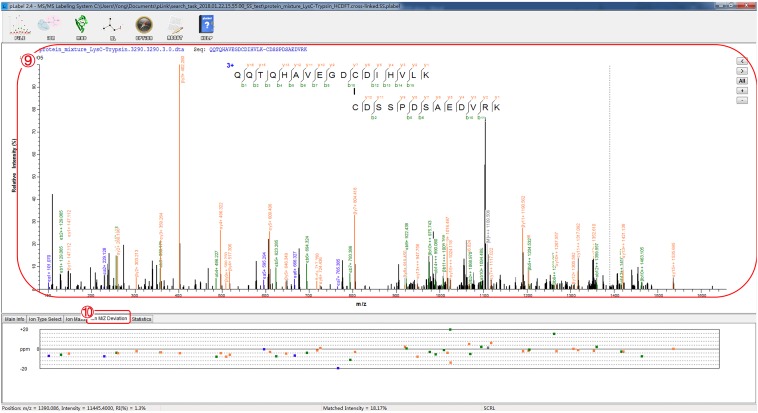
View matched ion peaks and mass deviations

### View matched ion of MS/MS spectra (Fig. 10)

(10) Inspect the spectrum to see how well ion peaks match with calculated peptide fragments.
(11) Select the tab “Ion M/Z Deviation” to see mass deviations of matched ion peaks.

## Result analysis

1. Rank the identification results by the cysteine sites. If a cysteine residue is found to form disulfide bonds with more than one cysteine residue, it could be a result of disulfide scrambling or false identification. Compare the *E* values, spectral counts, and intensities of these potentially conflicting identification results to try to distinguish a native disulfide bond from scrambled ones. This is based on the assumption that the native disulfide bond is the major form, so it should have higher signal intensity, higher spectral counts, and a smaller *E* value, which indicates a higher confidence in identification.
2. For purified proteins, such as pharmaceutical protein drugs, it is often required to map all the disulfide bonds in a protein. When some cysteines are missing from the disulfide identification results, one possibility is that they exist as free cysteines and the other is that they form disulfide bonds but these disulfide bonds have escaped identification. For the first possibility, one can expect free cysteines to be modified by NEM, so the corresponding modified linear peptides should be identifiable using conventional database search engines such as pFind. To find out whether the second possibility is true, we recommend reduction of disulfide bonds followed by alkylation and conventional database search to identify linear peptides; the cysteine-containing linear peptides can then be compared with those identified from the matched, non-reduced sample. In the case that a disulfide bond has escaped identification, first consider that the disulfide-containing peptide(s) may be too long or too short. Acting accordingly, choose proteases that will generate—concerning the cysteine residues in question—peptides of 6–20 (or better, 8–16) amino acids. Also, there may be an unknown modification near the missing disulfide bond. In this case, guess what it might be by sequence analysis with the help of modification prediction software, or use open search tools such as pFind 3.0 or Peaks to find possible modifications. Then, add the variable modification in pLink 2 search and see if it helps. Lastly, manual spectrum interpretation may help, but it requires a lot of time and experience.
**[? TROUBLESHOOTING]**
  Troubleshooting advice can be found in Table 3.

**Table 3:** Troubleshooting table

Problem	Possible reason	Solution
Many scrambled disulfide bonds are identified	pH is off; NEM gone bad after storage	Make sure that the pH of the urea buffer is 6.5; Make a fresh solution of NEM
The number of spectra of disulfide-linked peptides are too few	Insufficient digestion, masses of disulfide- linked peptides are too high; Over-digestion by non-specific proteases	Increase the amount of protease or the digestion time, or add another protease; Shorten the digestion time, try different time points
Certain disulfide bonds are not identified	Peptides are too long or too short, or too complex, *e.g.*, containing three peptides; Unexpected modifications on the disulfide-linked peptides	Digest the samples with different proteases; Try to identify the modification or treat with glycosylase and see what happens
pLink 2 report “MS1 or MS2 not completely extracted”	No MSFileReader installed	Install MSFileReader https://github.com/pFindStudio/pLink2/wiki/FAQ#how-to-install-msfilereader
Error in “loading label file”	“mgf” file is missing from the directory of the raw file	Don’t delete or move the “mgf” file. If it must be moved, change the path in “.plabel” file
